# Supplementary material for: Distribution of falciparum and non-falciparum malaria among symptomatic malaria patients in Dschang, West Region of Cameroon
Source: PLoS One. 2026 Feb 24;21(2):e0340824. doi: 10.1371/journal.pone.0340824 (PMC12931766; doi:10.1371/journal.pone.0340824)
Supplement: S2 Table — (DOCX) [file pone.0340824.s002.docx]

**Supplementary Table S2**: Multivariate logistic regression of sociodemographic factors (Gender, Age, level of education) associated with malaria infection.

|  | Malaria outcome | | |
| --- | --- | --- | --- |
| *Predictors* | *Odds Ratios* | *95 %CI* | *p.value* |
| Gender [Male] | 1.48 | 1.09 – 2.20 | 0.051 |
| Age [>49] | 0.69 | 0.32 – 1.45 | 0.327 |
| Age [15-49] | 0.78 | 0.37 – 1.61 | 0.509 |
| Age [6-14] | 1.16 | 0.39 – 3.60 | 0.797 |
| Level of education [secondary] | 1.32 | 0.67 – 2.63 | 0.420 |
| Level of education [Primary] | 1.38 | 0.88 – 2.42 | 0.980 |
| Level of education [University] | 0.81 | 0.49 – 1.32 | 0.391 |
